# Advances in direct detection of lysine methylation and acetylation by nuclear magnetic resonance using ^13^C-enriched cofactors

**DOI:** 10.1016/j.ymeth.2023.07.010

**Published:** 2023-07-29

**Authors:** Olivia A. Fraser, Kevin E.W. Namitz, Scott A. Showalter

**Affiliations:** a Center for Eukaryotic Gene Regulation, Department of Biochemistry and Molecular Biology, The Pennsylvania State University, University Park, PA 16802, United States; b Department of Chemistry, The Pennsylvania State University, University Park, PA 16802, United States

**Keywords:** Acetylation, Methylation, Nuclear magnetic resonance, Histone, Post-translational modification

## Abstract

Post-translational modifications (PTMs) are reversible chemical modifications that can modulate protein structure and function. Methylation and acetylation are two such PTMs with integral and well-characterized biological roles, including modulation of chromatin structure; and unknown or poorly understood roles, exemplified by the influence of these PTMs on transcription factor structure and function. The need for biological insights into the function of these PTMs motivates the development of a nondestructive and label-free method that enables pursuit of molecular mechanisms. Here, we present a protocol for implementing nuclear magnetic resonance (NMR) methods that allow for unambiguous detection of methylation and acetylation events and demonstrate their utility by observing these marks on histone H3 tail as a model system. We leverage strategic isotopic enrichment of cofactor and peptide for visualization by [^1^H, ^13^C]-HSQC and ^13^C direct-detect NMR measurements. Finally, we present ^13^C-labeling schemes that facilitate one-dimensional NMR experiments, which combine reduced measurement time relative to two-dimensional spectroscopy with robust filtering of background signals that would otherwise create spectral crowding or limit detection of low-abundance analytes.

## 1. Introduction

Translated proteins undergo an array of chemical modifications, referred to biochemically as post-translational modifications (PTMs) that enhance the functional diversity of proteins available in the cell [1,2]. Here we focus on lysine methylation and acetylation, for which the classical case is a diverse set of modifications to the N-terminal tails of histone proteins, which regulate chromatin structure by establishing the landscape of protein–protein interactions available to a given region of chromatin [3,4]. For example, trimethylation at histone H3 tail lysine 4 (H3K4me_3_), H3K36me_3_ and cumulative histone tail acetylation are all associated with transcriptional activity, while H3K9me_3_ is associated with transcriptional repression in facultative heterochromatin [5]. Dysregulation of histone methylation and acetylation are associated with human cancers, highlighting the necessity of biophysical characterization of marks to uncover molecular mechanisms that can be targeted therapeutically [6–8]. While histone H3 tail PTMs are relatively well-studied, thousands of proteins undergo acetylation and/or methylation, and deep mechanistic knowledge of the impacts of many of these modifications remains unknown [9,10]. Both this general observation and the specific case study of histone tail modification motivate development of novel, facile methods to study structure–function relationships involving modified lysine residues.

While not comprehensive, the following are a selection of methods currently available to observe protein methylation and acetylation. Radioisotope enriched cofactors can be used to monitor reaction progress, including ^3^H or ^14^C labeled S-adenosyl methionine (SAM) as the methyl-bearing cofactor for a lysine methyltransferase (KMT) or ^3^H or ^14^C labeled acetyl-Coenzyme A (acetyl CoA) as an acetyl-bearing cofactor for a lysine acetyltransferase (KAT) [11–13]. Spectrophotometric methods have been utilized to monitor deacetylation utilizing a synthetic peptide containing a fluorophore and quenching group, which become delocalized upon acetylation [14]. Methyltransferase activity can be coupled to NADPH oxidation for observation by spectrophotometry [15]. Accumulation of acetate or nicotinamide, depending on the histone deacetylase family being studied, has also been utilized to monitor deacetylation [16,17]. Free amine, a generic reaction product of deacetylation, regardless of the catalytic mechanism, can also be monitored using fluorescamine and fluorescence readout [18]. Mass spectrometric methods provide site-specific information, but are limited to discontinuous observation [19–21] Additionally, proton-detected nuclear magnetic resonance (NMR) experiments can be used for detection of methylation and acetylation [22]. In summary, an array of methods is available to study methyl and acetyl placement and removal. However, many are dependent on indirect readouts, while those which are direct are often unable to monitor reaction progress continuously or disrupt characterization of downstream, PTM-mediated complexes.

We have recently contributed novel NMR methods that provide unique opportunities for continuous, quantitative, and site-specific interrogation of structurally native lysine methylation [23] and acetylation [24]. Here, we provide comprehensive protocols for adoption of these technologies, with extensions to a previously unexplored ^13^C direct-detect format for methylation readout, and faster one-dimensional experiments that do not require homonuclear decoupling during detection for both methylation and acetylation. These experiments rely on isotopic enrichment of the cofactor, either SAM or acetyl CoA, with an NMR-visible nucleus (^13^C) for direct detection of the installed mark and so we provide detailed protocols for their preparation. To demonstrate our methods, we highlight the ease and utility of observing methylated and acetylated histone H3 tail. We foresee these experiments becoming valuable tools to monitor methylation and acetylation events due to their specificity, speed, and lack of perturbation to the native chemistry of the modified proteins.

## 2. Materials and methods

### 2.1. Rationale

To support the protocols described herein for *in vitro* enzymatic installation of methyl or acetyl groups on the peptide of interest, ^13^C enriched, and thus NMR visible, cofactors are required. Generating these cofactors enables detection of lysine methylation and acetylation by both [^1^H, ^13^C]-HSQC and ^13^C direct-detect experiments described in detail here (Table 1). While [^1^H, ^13^C]-HSQC has the benefit of high sensitivity, ^13^C direct-detect has the benefit of high selectivity; as such, the availability of these complementary methods extends detection capabilities to a wide variety of systems and applications.

**Fig. 1.**
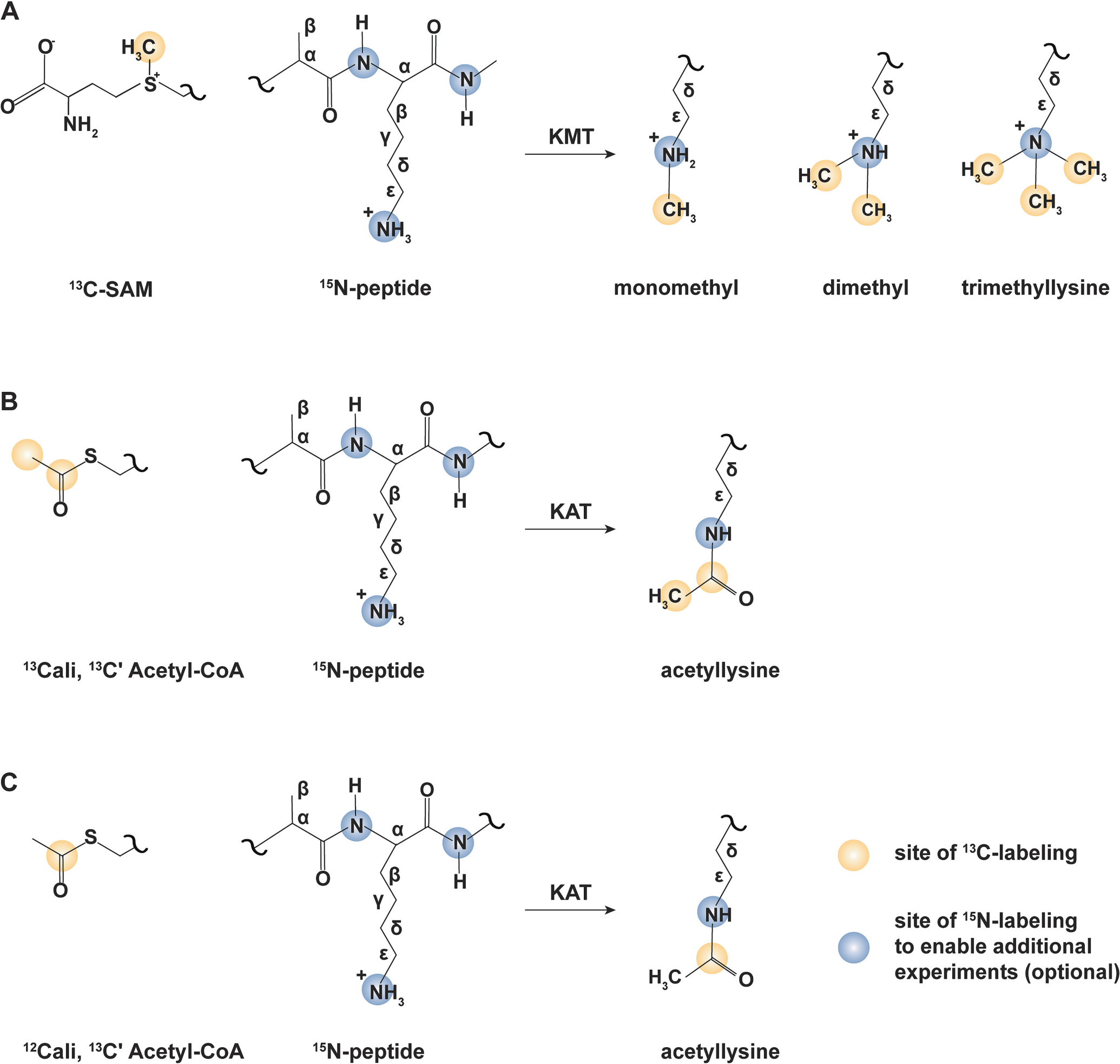
Isotope enrichment schemes to support ^13^C direct-detect NMR observation of lysine post-translational modification. (A)^13^C-methyl S-adenosyl methionine (SAM) serves as a cofactor to enzymatically transfer 1–3 methyl groups to substrate lysine residues. Uniform ^15^N enrichment of the peptide is required to support the reported [^15^N, ^13^C]-CaliN-K_me_ variants. (B) ^13^C_ali_, ^13^Cʹ -acetyl co-enzyme A serves as a cofactor to enzymatically transfer an acetyl group to substrate lysine residues. ^13^C direct-detect (carbon and proton-start [^13^C,^13^C] CaliCO-K_ac_ variants) can be used to observe acetyllysine without isotopic enrichment of peptide substrate. ^15^N-enrichment of peptide substrate enables the full suite of ^13^C direct-detect experiments (carbon and proton-start [^13^C,^15^N] CON-K_ac_-IPAP variants). Full isotopic enrichment of peptide substrate is permitted (methyl-selective [^13^C,^15^N] CON-K_ac_-IPAP) Schemes portrayed in A and B also permit observation by [^1^H, ^13^C]-HSQC. (C). ^12^C_ali_, ^13^Cʹ -acetyl co-enzyme A serves as a cofactor to enzymatically transfer an acetyl group to substrate lysine residues. Uniform ^15^N enrichment of the peptide is required to support the reported [^15^N, ^13^C]–CON-K_ac_ variants without IPAP virtual decoupling.

The general scheme for coupling ^13^C-enriched cofactors to (potentially isotopically enriched) substrate proteins in support of [^1^H, ^13^C]-HSQC and ^13^C direct-detect experiments is provided in Fig. 1. While only ^13^C-enriched cofactor is absolutely required for detection of both PTMs, coupling to ^15^N-enriched peptide substrate permits observation of carbon–nitrogen correlations and the isotope-filtered one-dimensional experiments described herein. Rather than relying extensively on chemical synthesis, which often lacks the site-specificity of enzymatic catalysis, the protocols described rely on recombinant expression of lysine-modifying enzymes. Thus, we first describe protocols to produce necessary reaction components. Next, we describe state-of-the-art protocols for implementing NMR detection of methylation and acetylation, including novel experiments (2D and 1D [^15^N, ^13^C]-CaliN-K_me_ and [^15^N, ^13^C]–CON-K_ac_ IPAPless experimental variants). Demonstration data are provided from methylation and acetylation of the histone H3 N-terminal tail (residues 1–44, containing an exogenous C-terminal tryptophan residue) using ^13^C-SAM and either ^13^Cʹ/^13^C_ali_ or ^13^Cʹ/^12^C_ali_ acetyl-CoA as the respective cofactors. We demonstrate our technique through observation of monomethylation, using recombinantly expressed SETD7, and acetylation using recombinantly expressed SAGA components ADA2 and GCN5 (GCN5/ADA2) for enzymatic acetyl transfer.

### 2.2. Reagent preparation

#### 2.2.1. Plasmids

The plasmid (pET3a) coding for the *Homo sapiens* Histone H3 tail (residues 1–44) was a gift from the lab of Catherine Musselman, University of Colorado Anschutz [25]. An exogenous C-terminal tryptophan residue was added to constructs used here for the purpose of quantification. The plasmid coding for the *Escherichia coli* codon-optimized *H. sapiens* histone-lysine N-methyltransferase SETD7 (residues 109–366, referred to simply as SET7 from here on) with an exogenous preceding Met residue and non-cleavable 6X His-tag was obtained from Addgene (Addgene #: 40746; Watertown, MA). This plasmid was altered to create full-length SET7 by Gibson assembly, using a gBlock consisting of codons for residues 1–108 (codon-optimized for *E. coli*). The coding region of thermophilic Archaea *Methanocaldococcus janaschii* SAM synthetase (Mj SAM synthetase) in the pET19b vector was a gift from the lab of Squire Booker, Pennsylvania State University. SAGA components Ada2 and Gcn5 (GCN5/ADA2) were expressed from the polycistronic vector pST44-HISMBPNyAda2t5-yGcn5t10x2, which was a gift from the lab of Song Tan, Pennsylvania State University (Addgene #64007, [26–28]).

#### 2.2.2. Protein expression and purification

##### 2.2.2.1. Histone H3 Tail, (1–44 with exogenous Trp).

A 5-mL culture of BL21(DE3) *Fhu2A*^−^ *E. coli* transformed with the H3 (1–44) plasmid was grown in Luria broth (LB) containing 100 μg/mL ampicillin at 37 °C for between 8 and 10 h. 500 μL of turbid 5-mL culture was used to inoculate 50 mL of M9 minimal media supplemented with 1 g/L ^15^N-ammonium chloride (Cambridge Isotope Laboratories, DNLM-8739-PK), 3 g/L of ^12^C_6_-glucose, 1X MEM vitamins (Corning), 1 mM MgSO_4_, 100 μg/mL ampicillin and 1X trace metals (Teknova, T1001). Growth proceeded for 10 h to a typical optical density at 600 nm (OD_600_) of 2.5–3.5. Subsequently, 1L of the M9 media supplemented as described was inoculated with the turbid 50 mL culture to an initial OD_600_ of 0.05–0.1 and grown at 37 °C to an OD_600_ of 0.9. Expression was initiated with 0.4 mM isopropyl β-D-1-thiogalactopyranoside (IPTG). Following a 3-hour expression at 37 °C, the cells were harvested by centrifugation at 3,900 *xg* at 4 °C. The pellet was washed with 35 mL of 20 mM Tris, 20 mM NaCl, 2 mM EDTA, pH 7.5 and stored at −80 °C.

Cells containing recombinantly expressed H3 (1–44) were lysed by sonication in 35 mL total volume of 50 mM Tris pH 9.0, 100 mM NaCl, 1 mM phenylmethylsulfonyl fluoride and 100 μL protease inhibitor cocktail set V, EDTA-free (Millipore Sigma) (50% duty cycle, 50% amplitude). Lysed cells were cleared by centrifugation at 14,000 *xg* for 30 min at 4 °C. The clarified supernatant was held at 80 °C in a water bath for 3 min, transferred for incubated on ice until reaching a temperature of 20 °C, and held on ice for two additional minutes. The product was clarified by centrifugation at 14,000 *xg* for 25 min at 4 °C.

The supernatant was filtered with a 0.45 μm syringe filter before addition to an SP sepharose gravity column equilibrated with 50 mM Tris pH 9.0, 100 mM NaCl. After loading, the column was washed with 1 column volume (CV) of the equilibration buffer, followed by 50 mM Tris pH 9.0, 100 mM NaCl, 3 M Urea (2CV), and 50 mM Tris pH 9.0, 100 mM NaCl, 6 M Urea (2 CV). The protein was eluted using the aforementioned buffer containing a gradient of 500–1000 mM sodium chloride in 100 mM increments (1 CV/fraction), where the 700 mM fraction contained the protein of interest. The elution fraction was confirmed by SDS-PAGE (Tris-Tricine, 16%), with both 2,2,2-trichloroethanol and Coomassie stain, and subsequently exchanged using a 3 kDa MWCO Amicon centrifugal filter (UFC9003) to 50 mM potassium phosphate, pH 7.2, 150 mM potassium chloride for storage. Protein was quantified by absorbance of a dilution series at 280 nm (ε_H3W_ = 6990 M^−1^cm^−1^). Expected yield from purification of protein recombinantly expressed in 1 L of minimal media is 1.5 to 2 mg.

##### 2.2.2.2. Expression and purification of Mj SAM synthetase.

A 50-mL culture of BL21 pLysS (*Fhu2A-*) DE3 cells transformed with the Mj SAM synthetase expression vector was grown in LB supplemented with 100 μg/mL ampicillin at 37 °C with 200 RPM shaking for 14–16 h. 20 mL of turbid culture was used to inoculate 3L of LB media, supplemented with 100 μg/mL ampicillin. Growth proceeded at 37 °C with 150 RPM shaking for 2–3 h, until the OD_600_ of the cultures was between 0.8 and 0.9. The cultures were then induced with a final concentration of 0.5 mM IPTG. Following a 3-hour expression at 37 °C, the cells were harvested by centrifugation at 3,900 *xg* at 4 °C. The pellet was washed with fresh LB medium and stored at −80 °C.

Cells containing recombinant protein were thawed on ice, resuspended with 150 mL 100 mM HEPES, pH 7.5; 300 mM KCl; 5% (v/v) glycerol; 2 mM TCEP-HCl supplemented with 150 μL Triton X-100, 150 mg Lysozyme, 1 mM phenylmethylsulfonyl fluoride and 15 mg DNase I, and stirred for 30 min. Cells were lysed by sonication (50% duty cycle, 50% amplitude, 3 min on) for six repetitions. The sample was heat-shocked at 75 °C for 1 h and cleared by centrifugation at 21,000 *xg* for 1 h at 4 °C. The supernatant was decanted and applied to a Ni^2+^-NTA column (G-Biosciences) at room temperature that was previously equilibrated with 100 mL 100 mM HEPES, pH 7.5; 300 mM KCl; 5% (v/v) glycerol; 2 mM TCEP-HCl. Once loaded, the resin was washed with 100 mL of 100 mM HEPES, pH 7.5; 300 mM KCl; 5% (v/v) glycerol; 2 mM TCEP-HCl. The retained protein was eluted in 60 mL of 100 mM HEPES, pH 7.5; 150 mM KCl; 250 mM Imidazole; 10% (v/v) glycerol; 4 mM TCEP-HCl. The protein was exchanged into 100 mM Tris, pH 8.0; 50 mM KCl; 10% (v/v) glycerol; 4 mM TCEP-HCl using an Amicon 10 K MWCO centrifugal filter. The sample was then concentrated, divided into 1 mL aliquots, and frozen at −80 °C for further use.

##### 2.2.2.3. SET7 lysine methyltransferase.

BL21(DE3) *Fhu2A*^−^ cells transformed with the plasmid encoding full-length SET7 were grown at 37 °C on LB medium supplemented with 50 μg/mL Kanamycin. A single colony was used to inoculate LB medium (100-mL, 50 μg/mL Kanamycin) which was grown at 37 °C until turbid. LB medium (3L, 50 μg/mL Kanamycin) was inoculated with 40-mL of turbid overnight culture. Growth proceeded at 37 °C until reaching an OD_600_ of 1.0. Expression was initiated with 0.5 mM IPTG and proceeded for 20 h at 15 °C with shaking at 180 RPM. To harvest, the cells were centrifuged at 3,900 *xg* at 4 °C and stored at −80 °C.

Cells containing recombinant protein were thawed on ice, resuspended with 50 mM Tris, pH 7.5; 500 mM NaCl; 20 mM Imidazole; 3 mM β-mercaptoethanol, supplemented with 1 mM phenylmethylsulfonyl fluoride, and 100 μL protease inhibitor cocktail V. Cells were lysed by sonication (50% duty cycle, 50% amplitude, 3 min on) for six repetitions. The cell lysate was cleared by centrifugation at 14,000 x*g* for 1 h at 4 °C. The supernatant was applied to a 30-mL bed volume Ni^2+^-NTA column (G-biosciences) at room temperature previously equilibrated with 50 mM Tris, pH 7.5; 500 mM NaCl; 20 mM Imidazole; 3 mM β-mercaptoethanol (2 CV). Once loaded, the resin was washed with 50 mM Tris, pH 7.5; 500 mM NaCl; 20 mM Imidazole; 3 mM β-mercaptoethanol; 0.1% (v/v) Triton X-100 (3 CV). The retained protein was eluted in 50 mM Tris, pH 7.5; 500 mM NaCl; 500 mM Imidazole; 3 mM β-mercaptoethanol (1 CV). The protein was exchanged into 50 mM Tris, pH 7.5; 150 mM NaCl; 5 mM DTT using an Amicon 10 K MWCO centrifugal filter. The sample was concentrated to 1 mM, divided to single use aliquots, and stored at −80 °C.

##### 2.2.2.4. GCN5/ADA2 acetyltransferase.

A 5-mL culture of BL21(DE3) *Fhu2A*^−^ *E. coli* transformed with the GCN5/ADA2 plasmid was grown at 37 °C in LB medium supplemented with 50 μg/mL ampicillin. A 5-mL turbid culture (OD_600_ = 2.5–3.5) was used to inoculate 1L of LB medium. Growth proceeded at 37 °C until reaching an OD_600_ of 0.2. The growth temperature was adjusted to 18 °C and growth proceeded until an OD_600_ of 0.8–1.0 was reached. Expression was initiated with 0.5 mM IPTG and proceeded for 16–18 h at 18 °C. To harvest, the cells were centrifuged at 3,900 *xg* at 4 °C and stored at −80 °C.

Cells containing recombinant protein were lysed by sonication (50% duty cycle, 50% amplitude, 3 min on) in 50 mM sodium phosphate pH 7.0, 300 mM NaCl, 10 mM imidazole, 5 mM β-mercaptoethanol,1 mM phenylmethylsulfonyl fluoride. The cell lysate was cleared by centrifugation for 30 min at 14,000 *xg* and 4 °C. His-tagged protein was bound to Ni^2+^-NTA resin, washed with 50 mM sodium phosphate pH 7.0, 300 mM sodium chloride, 10 mM imidazole, 5 mM β-mercaptoethanol (2 CV) and eluted with 50 mM sodium phosphate pH 7.0, 300 mM sodium chloride, 100 mM imidazole, 5 mM β-mercaptoethanol (1.5 CV). 6x His TEV protease was used to cleave purification tags from recombinant protein while dialyzing against 50 mM sodium phosphate pH 7.0, 300 mM sodium chloride, 10 mM imidazole, 5 mM β-mercaptoethanol for 48 h at 4 °C. TEV was removed through an additional Ni^2+^-NTA binding step, where GCN5/ADA2 was captured in the flow through. GCN5/ADA2 was buffer exchanged to 50 mM sodium phosphate pH 7.0, 10 mM imidazole, 5 mM β-mercaptoethanol and supplemented with 20% glycerol for storage in single use aliquots at −80 °C.

#### 2.2.3. Generation of ^13^C-enriched enzyme cofactors

##### 2.2.3.1. Synthesis and purification of ^13^C SAM.

In preparation for SAM synthesis the following stocks were separately made in distilled deionized water: 100 mM ^13^C-methionine (Cambridge Isotope Laboratories, CLM-206), 150 mM ATP, 10X reaction buffer (500 mM Tris, pH 8.0, 500 mM KCl, 500 mM MgCl_2_), and 500 mM β-mercaptoethanol. The reaction to produce SAM was carried out by mixing 800 μL ^13^C-methionine, 800 μL ATP, 800 μL reaction buffer, 48 μL β-mercaptoethanol, 4 mL of 200 μM SAM synthetase, and sufficient water to reach a final reaction volume of 8 mL. The reaction was allowed to progress at room temperature (typically 21 °C) for 5 h. Reaction progress was monitored by ^1^H NMR, which demonstrated that approximately 50% of the methionine was converted enzymatically to SAM in a typical reaction. The reaction was quenched with 500 uL of fuming HCl. The quenched reaction products were passed through a 0.22 μm syringe filter, diluted 1:5 with 1 mM sodium acetate, pH 5.0 and stored at 4 °C in one ~ 40 mL aliquot. Note that the reaction product can be stably stored for future purification at −80 °C after this step.

To begin purification of the ^13^C-SAM, a 90 mL carboxymethyl column (Sigma-Aldrich: CCF100) was charged with 2CV of 1 M sodium acetate, pH 5.0 and equilibrated with 2 CV of 1 mM sodium acetate, pH 5.0. The reaction products from SAM synthesis were thawed and applied to the equilibrated CM column, washed with 2 CV of 1 mM sodium acetate, pH 5.0, and eluted with 1 CV of 100 mM sulfuric acid. In our experience, the sulfuric acid solution helps stabilize the SAM molecule against undesired methyl transfer and (S, S) to (R, S) racemization. The elution fraction was dried using vacuum centrifugation to a total volume of 5 mL. The concentration of eluted ^13^C-SAM was approximated by absorbance at 259 nm, ε = 15,400 M^−1^, cm^−1^ (A_259_). In preparation for the next column, an aliquot containing approximately 1500 μg of SAM was prepared by mixing 1:1 with 10 mM HCl; for a typical SAM preparation, this required a 250 μL aliquot of the SAM stock. The quantity of SAM prepared in the working aliquot was calibrated against the observed productive binding capacity of the size exclusion column. The remainder of the SAM stock was stored with no observed degradation at −80 °C until future use.

The ^13^C-SAM aliquot advanced through further purification was diluted 1:2 with 10 mM HCl and applied to a P2 (Bio-Rad 90-mL) column previously equilibrated in 10 mM HCl. The column was washed with 10 mM HCl and 1.8 mL fractions were collected between 45 and 150 mL. Desalted ^13^C-SAM eluted between 75 and 100 mL based on conductivity and absorbance at 254 nm. Fractions containing desalted ^13^C-SAM were dried by vacuum centrifugation and subsequently combined to yield 100 μL total volume in 10 mM HCl.

SAM was quantified using A_259_ and direct NMR intensity comparison with DSS. For this quality control step, an NMR sample was prepared with 500 μM reaction product (estimated by A_259_), 500 μM ^12^C- trimethylsilylpropanesulfonate (DSS) in D_2_O. A ^1^H-1D was collected on a Bruker Avance AVIII 500 with TCI Cryoprobe using 32 k data points, 16 scans, a sweep width of 12 ppm, and a spectral center of 4.69 ppm (Bruker library pulse sequence *zgesgp* without decoupling, or *zgig* for the ^13^C-decoupled spectrum). Based on quantification from multiple column runs, a yield of approximately 750 μg highly active and desalted SAM can be expected.

##### 2.2.3.2. Synthesis and purification of ^13^C-Acetyl Coenzyme A.

Previously, we have reported the synthesis of ^13^C-Acetyl Coenzyme A with ^13^C-labeling on both the aliphatic and carbonyl carbon of the acetyl group, provided from 1,1′,2, 2′−^13^C acetic anhydride (Cambridge Isotope Laboratories, CLM-1161). To eliminate the need for homonuclear decoupling in 1D NMR experiments here, ^13^C-Acetyl Coenzyme A with specific labeling of the carbonyl position was generated using a similar protocol and 1,1′-^13^C_2_ acetic anhydride (Cambridge Isotope Laboratories, CLM-1159) as the acetyl source. In brief review of the synthetic protocol, CoA lithium salt (1 equivalent, CoALA Biosciences, AC02) and acetic anhydride with the desired ^13^C-labeling (1.8 equivalents) were combined in 200 μL 0.5 M sodium bicarbonate in a capped recovery vessel. The reaction proceeded while incubating on ice for 45 min [24,29]. The yield of acetyl-CoA is generally > 95% and unreacted acetate does not inhibit downstream enzymatic reactions. Therefore, the reaction product was used without further purification.

For quantitation and quality control, an aliquot of 20 μL reaction product was combined with 1 mM DSS and brought to 500 μL with D_2_O. A ^1^H-1D NMR spectrum was collected on a Bruker Avance AVIII 500 with TCI Cryoprobe using 32 k data points, 16 scans, a sweep width of 12 ppm, and a spectral center of 4.69 ppm (Bruker library pulse sequence *zgesgp*).

### 2.3. In vitro reactions to generate modified lysine products

#### 2.3.1. Methylation

The peptide substrate (^15^N-H3, 500 μM) was added to ^13^C-SAM (1 mM), 1 mM DSS, 50 mM Tris (pH 8.5), 50 mM NaCl, 1 mM TCEP, 1% D2O (v/v). 100 μM SET7 was added to initiate the reaction, to a total volume of 500 μL. The reaction proceeded at 25 °C for 14 h. The reaction was monitored in a high quality 5 mm NMR tube with no further processing.

#### 2.3.2. Acetylation

^13^C_ali_, ^13^Cʹ-Acetyl CoA or ^12^C_ali_, ^13^Cʹ-Acetyl CoA (2.1 mM), were combined in 50 mM Tris/50 mM BisTris, 100 mM sodium acetate, 1 mM DTT, pH 7.5. The peptide substrate (^15^N-H3, 1 mM) was added to the resultant solution. Here, 2.6 μM GCN5/ADA2 was added to initiate the reaction, to a total volume of 500 μL. The reaction proceeded at 25 °C for 3 h. The reaction was quenched by heating at 85 °C for 3 min, followed by centrifugation at 12,500 *xg* for 10 min. The supernatant was buffer exchanged to 50 mM potassium phosphate, pH 7.2; 150 mM potassium chloride. DSS (1 mM) and D2O (5%) were added to bring the solution to a final volume of 500 μL and transferred to a high quality 5 mm NMR tube.

### 2.4. Nuclear magnetic resonance detection of PTM

#### 2.4.1. General parameters for NMR data acquisition

All NMR experiments were conducted on either a Bruker Avance NEO 600 MHz spectrometer or a Bruker Avance III 500 MHz spectrometer, each equipped with a 5 mm TCI cryoprobe. The typical 90° ^1^H hard pulse was 12–14 μs and the typical ^13^C 90° hard pulse was 15 μs. All pulsed field gradients were applied for 1 ms with a sine shape. All spectra were recorded at 25 °C.

#### 2.4.2. [^1^H, ^13^C]-HSQC based detection

1D-^1^H experiments and [^1^H, ^13^C]-HSQC were collected using the standard pulse programs from the Bruker Topspin library. 1D-^1^H experiments were collected with 32 k data points, 16 scans, and a sweep width of 16 ppm centered at 4.69 ppm with a recycle delay of 1 s. [^1^H, ^13^C]-HSQC experiments were collected with 1024 direct data points and 128 real indirect data points, 16 scans of signal averaging, and a recycle delay of 1 s. Spectra were acquired with a sweep width of 2.98 ppm centered at 2.95 ppm in the direct dimension and a sweep width of 70 ppm centered at 40 ppm in the indirect dimension.

#### 2.4.3. Observation of methyllysine by ^13^C direct-detect NMR

**Fig. 2.**
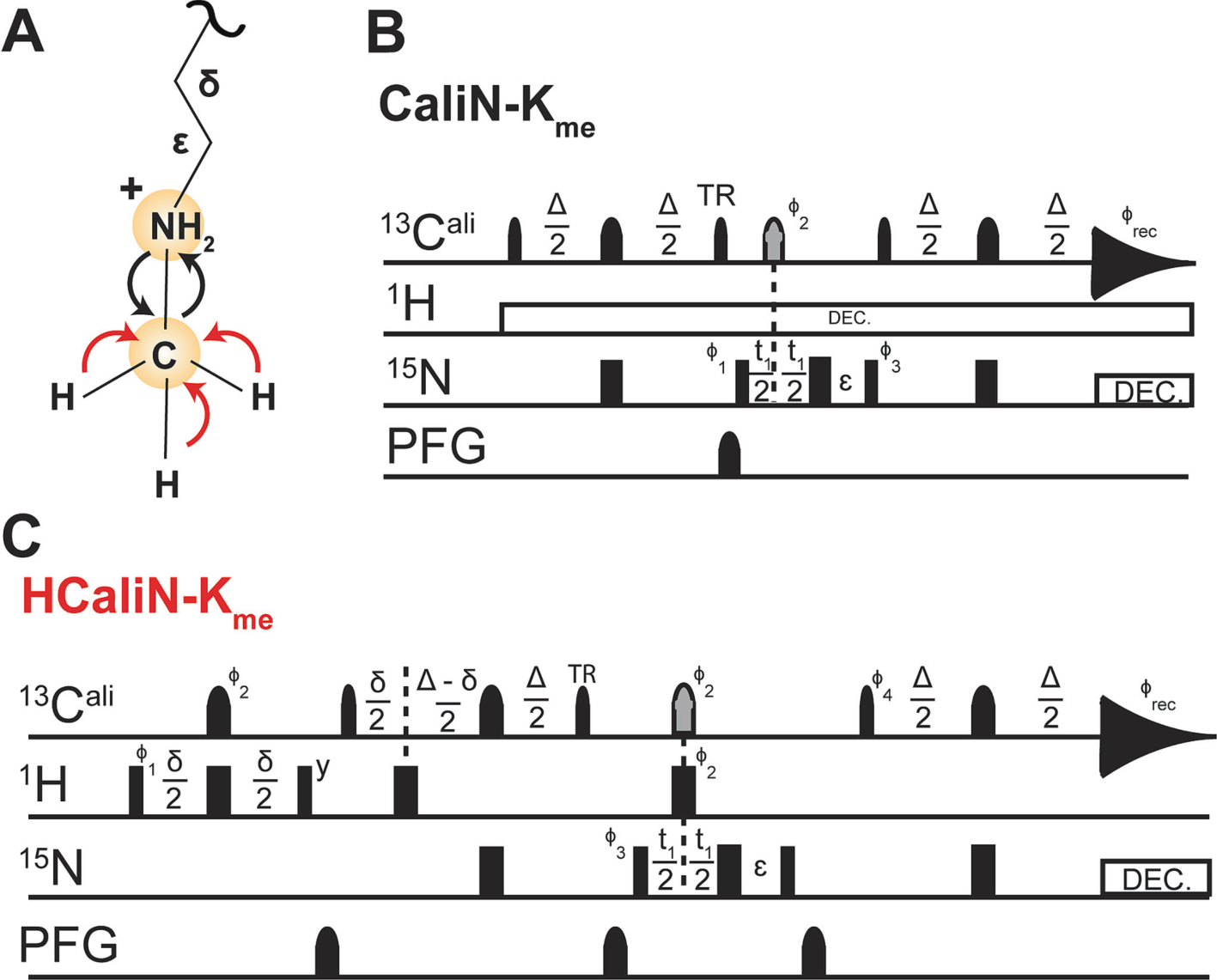
A visual schematic and the timing diagrams for the ^13^C direct-detect experiments used to observe methyllysine. (A) A schematic of the magnetization transfer pathway is shown on monomethyllysine. The carbon-start [^13^Cali,^15^N]-CAliN-K_me_ is shown in black arrows. The initial INEPT transfer step between methyl proton and carbon in the methyl proton-start [^13^Cali,^15^N]-CAliN-K_me_ is displayed as a red arrow. (B) Pulse sequence for the carbon-start [^13^Cali,^15^N]-CAliN-K_me_ experiment. The delay times are Δ = 9.8 ms, Δ_1_ = 25 ms, and ε = t_1_(0) + pC180. The phase cycle is φ_1_ = x,−x; φ_2_ = x,x,−x,−x; φ_3_ = 4(x),4(−x); φ_REC_ = x,− x, x, −x, −x, x, −x, x. (C) Pulse sequence for the methyl proton-start [^13^Cali,^15^N]-CAliN-K_me_ experiment. The delay times are *δ* = 1.44 ms, Δ = 9.8 ms, and ε = t1(0) + pC180. The phase cycle is φ_1_ = x,−x; φ2 = 8(x),8(−x); φ3 = x,x,−x,−x; φ4 = 4(x),4(−x); φREC = x,− x, −x, x, −x, x, x, −x. Quadrature detection in the indirect dimension is obtained by States-TPPI incrementation of φ_3_. For both pulse sequences, narrow and wide rectangular pulses correspond to 90° and 180° hard pulses, respectively. All ^13^C pulses are represented with narrow (90°, Q5_sebop) shapes and wide (180°, Q3_surbop) shapes. The gray pulse on ^13^C indicates a band-selective ^13^Cʹ and ^13^C_ali_ inversion pulse. The ‘TR’ label indicates a time-reversed 90° pulse. All pulses are applied with x-phase unless otherwise indicated. Pulsed field gradients (Gz) are also represented by shapes. Nitrogen decoupling is achieved with garp4 (220 μs, 2.367 W), and proton decoupling is achieved using waltz65 (70 μs, 0.096 W). ^15^N chemical shift evolution is measured during t_1_, with quadrature achieved by States-TPPI incrementation of φ_3_. In the 1D variant, t_1_ is held constant at the time of the first increment (3 μs) and is not collected as a variable delay. in both experiments, the carrier position is set to ^13^C_ali_, centered at 33 ppm. In the 1D variant of each pulse sequence, t_1_ is held constant at the time of the first increment (3 μs) and is not collected as a variable delay.

Direct NMR observation of lysine methylation can be achieved using the new ^13^C direct-detect [^15^N, ^13^C]-CaliN-K_me_ described herein. Inspiration for the 2D and 1D [^15^N, ^13^C]-CaliN-K_me_ came from the protonless CAN [30]. Key changes to this pulse sequence include movement of the spectral center and shaped pulses to a frequency suitable for aliphatic carbons and removal of the IPAP scheme required in the original experiment to support virtual decoupling in the ^13^C dimension. Inspiration for the methyl proton start [^15^N, ^13^C]-CaliN-K_me_ came from the 3D HCAN [30]. Timing diagrams for both experimental variants are provided in Fig. 2. 2D experiments displayed here were recorded with 1,024 direct data points and 128 indirect data points, 16 scans, and a sweep width of 33 ppm with a Center of 33 ppm in the direct dimension, and a sweep width of 70 ppm with a Center of 127 ppm in the indirect dimension.

Lysine methylation has also been observed by ^13^C 1D NMR (Bruker library pulse sequence *zgpg30*, parameter set C13CPD). The experiment displayed here was recorded with 32 k data points, 1024 scans, a sweep width of 236.6 ppm, and a center of 100 ppm.

#### 2.4.4. Observation of acetyllysine by ^13^C direct-detect NMR

Multiple experimental strategies are available for ^13^C direct-detect observation of acetyllysine, depending on user needs. Lysine acetylation utilizing ^13^Cali, ^13^Cʹ acetyl CoA cofactor can be directly observed through CaliCO-K_ac_-IPAP and CON-K_ac_-IPAP [24] or using the new 2D and 1D [^15^N, ^13^C]–CON-K_ac_ utilizing ^12^Cali, ^13^Cʹ acetyl CoA cofactor as described herein. Note that use of CON-based pulse sequences requires ^15^N-enrichment of the protein substrate. These strategies were inspired by the previously reported [^13^C,^13^C]-CACO and [^13^C,^15^N]–CON experiments conventionally used to monitor correlations in the peptide backbone [30]. ^13^C direct detect experiments utilizing ^13^Cali, ^13^Cʹ acetyl CoA cofactor require that steps be taken to enable homonuclear decoupling in the ^13^C direct dimension. This was achieved through in-phase anti-phase (IPAP) data acquisition and virtual decoupling in post-acquisition processing [31] In the Topspin data processing environment, virtual decoupling is achieved through application of the “splitcomb” processing script provided by Bruker. In both the [^13^C, ^13^C]-CaliCO-K_ac_-IPAP and [^15^N, ^13^C]–CON-K_ac_-IPAP, the ^13^C_ali_ – selective pulses are centered on 25 ppm. The representative [^13^C, ^13^C]-CaliCO-K_ac_-IPAP spectrum presented here was recorded with 1,024 direct data points and 128 indirect data points, 64 scans, and a sweep width of 20 ppm centered on 172 ppm in the direct dimension (Cʹ) and a sweep width of 60 ppm centered at 25 ppm in the indirect dimension (C_ali_). The representative [^13^C,^15^N]–CON-IPAP spectrum presented here was recorded with 1,024 direct data points and 256 indirect data points, 32 scans, and a sweep width of 20 ppm centered on 172 ppm in the direct dimension (Cʹ) and a sweep width of 42 ppm centered on 127 ppm in the indirect dimension (^15^N).

**Fig. 3.**
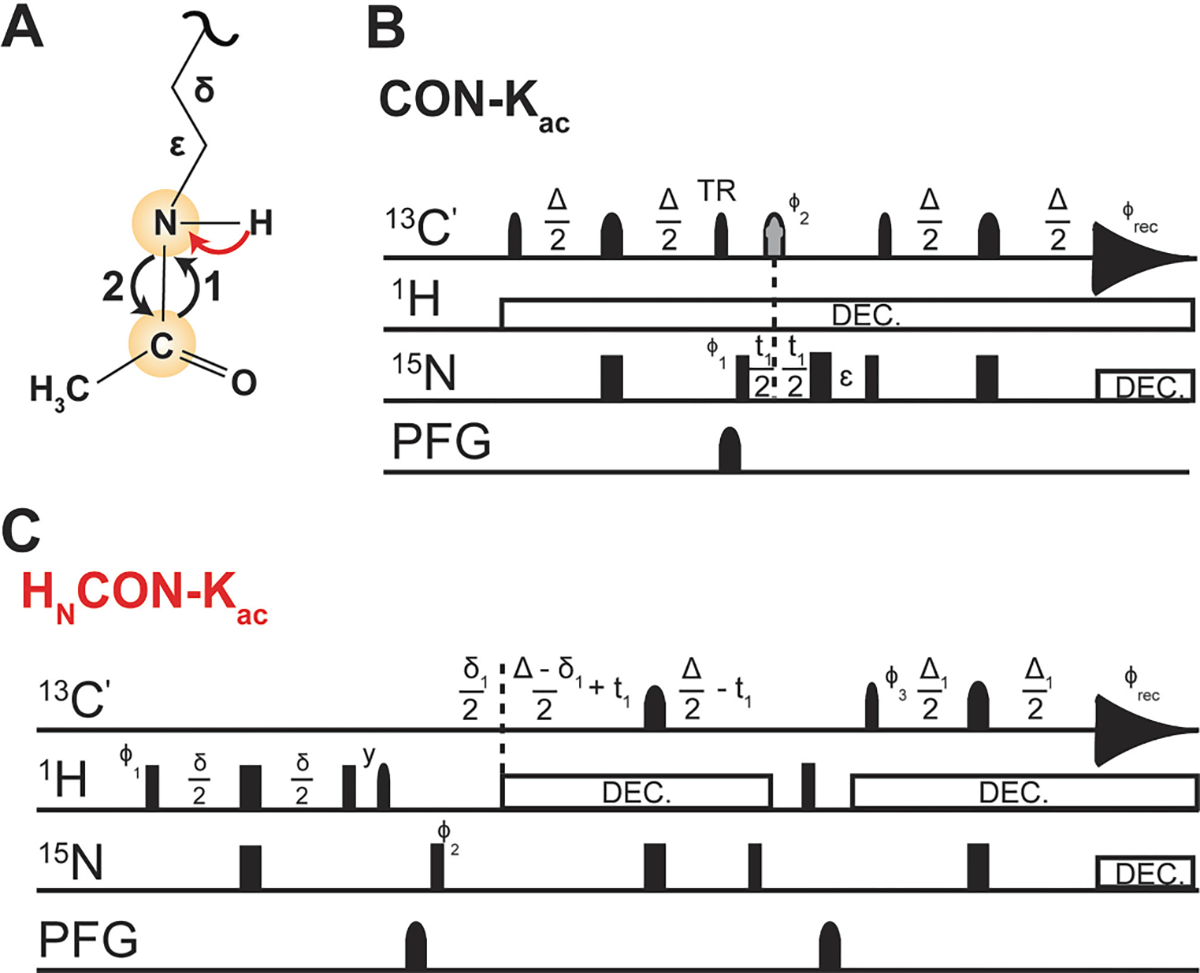
A visual schematic and the timing diagrams for the ^13^C- direct -detect experiments used to detect acetyllysine. (A) A schematic of the magnetization transfer pathway is shown on acetyllysine. The carbon-start [^13^Cʹ,^15^N]–CON-K_ac_ is shown in black arrows. The initial INEPT transfer step between amide proton and carbon in the amide proton-start [^13^Cʹ,^15^N]–CON-K_ac_ is displayed as a red arrow. (B) Pulse sequence for the carbon-start [^13^Cʹ,^15^N]–CON-K_ac_ experiment. The delay times are Δ = 9.8 ms, Δ_1_ = 25 ms, and ε = t1(0) + pC180. The phase cycle is φ_1_ = x,−x;φ_2_ = x,x,−x,−x; φ_3_ = 4(x),4(−x); φ_REC_ = x,− x, x, −x, −x, x, −x, x. ^15^N chemical shift evolution is measured during t_1_, with quadrature achieved by States-TPPI incrementation of φ_3_. (C) Pulse sequence for the amide proton-start [^13^Cʹ,^15^N]–CON-K_ac_ experiment. The delay times are *δ* = 4.6 ms, *δ*_1_ = 5.1 ms, Δ = 24.8 ms, Δ_1_ = 9.0 ms. The phase cycle is φ_1_ = x,−x; φ_2_ = x,x,−x,−x; φ_3_ = −y; φ_4_ = 4(x),4(−x); φ_REC_ = x,− x, −x, x, −x, x, x, −x. Quadrature detection in the indirect dimension is obtained by States-TPPI incrementation of φ_2_. For both pulse sequences, narrow and wide rectangular pulses correspond to 90° and 180° hard pulses, respectively. All ^13^C pulses are represented with narrow (90°, Q5_sebop) shapes and wide (180°, Q3_surbop) shapes. The gray pulse on ^13^C indicates a band-selective ^13^Cʹ and ^13^C_ali_ inversion pulse. The ‘TR’ label indicates a time-reversed 90° pulse. All pulses are applied with x-phase unless otherwise indicated. Pulsed field gradients (Gz) are also represented by shapes. Nitrogen decoupling is achieved with garp4 (240 μs, 2.81 W), and proton decoupling is achieved using waltz65 (80 μs, 0.209 W). In the 1D variants, t_1_ is held constant at the time of the first increment (3 μs) and is not collected as a variable delay.

Use of ^12^Cali, ^13^Cʹ acetyl CoA cofactor permits collection of the novel 2D and 1D [^15^N, ^13^C]–CON-K_ac_ reported here. Inspiration came from the protonless CON and the aforementioned past observation of ^13^C_ali_, ^13^Cʹ labeled acetyllysine [24,30]. However, the necessity for virtual decoupling in the direct ^13^C dimension is removed due to strategic labeling of the acetyllysine at the carbonyl position alone. Timing diagrams for both experimental variants are provided in Fig. 3. 2D experiments displayed here were recorded with 1,024 direct data points and 100 indirect data points, 16 scans, and a sweep width of 44 ppm with a Center of 173.5 ppm in the direct dimension, and a sweep width of 44.8 ppm with a Center of 119 ppm in the indirect dimension.

#### 2.4.5. Observation of methyllysine and acetyllysine by 1D ^13^C direct-detect

The ^13^C direct-detect variants used to detect methyllysine, the [^15^N, ^13^C]-CaliN-K_me_ and the methyl proton start [^15^N, ^13^C]-CaliN-K_me_, can be performed in 1D format. 1D acquisition yields the ^13^C_ali_ chemical shift with excellent suppression of background signal due to the isotope filtering scheme, which can simplify analysis compared to traditional [^1^H, ^13^C]-HSQC methods while maintaining the advantages associated with a short acquisition time. The 1D experiments displayed here were recorded with a sweep width of 33 ppm and a spectral center of 33 ppm. 1D experiments were collected with 4,096 data points and 128 scans.

Similarly, for acetyllysine, the IPAPless carbon and amide start [^15^N, ^13^C]–CON-K_ac_ variants can be performed as 1D experiments to further shorten their acquisition time. 1D acquisition yields the ^13^Cʹ chemical shift of the acetyllysine while suppressing background from natural abundance ^13^Cʹ present in solution. 1D experiments were recorded with 8,192 data points, 256 scans, a spectral center of 173.5 ppm and a sweep width of 33 pm.

#### 2.4.6. Data processing

On-instrument processing of all spectra was performed using Bruker Topspin V 4.0.5. Viewing and processing for image generation of all 2D spectra were carried out in NMRFAM-SPARKY [32].

## 3. Results and discussion

Post translational modifications (PTMs), including methylation and acetylation, can have dramatic effects to protein function, thus necessitating their biophysical characterization. One well-studied example includes the methylation and acetylation of histone protein N-terminal tails. For example, histone H3 trimethylation at lysine 4, and acetylation on multiple lysine residues, are associated with transcriptional activity [5] Nuclear magnetic resonance (NMR) has proven a valuable technique to investigate PTM-mediated effects as it preserves sidechain chemistry and can be used as a continuous technique for monitoring real-time installation of marks.

### 3.1. Commentary on reagent generation

The methods presented here provide strategies for isotopic enrichment of KMT and KAT cofactors with strategically placed spin ½ nuclei for peptide modification (^13^C S-adenosyl methionine; ^13^C_ali_, ^13^Cʹ-Acetyl CoA; or ^12^C_ali_, ^13^Cʹ-Acetyl CoA). Isotopic enrichment of peptide, while not necessary, enables access to the full suite of ^13^C direct-detect experiments described. To empower users to utilize this technique, we provide commentary on design choices for cofactor and peptide substrates and insights into the protocol steps that have the greatest impact on final yield and spectral quality.

Here, ^13^C S-adenosyl methionine (SAM) was used as the cofactor for enzymatic installation of a methyl group onto H3 peptide substrate. The detailed protocol for enzymatic synthesis and purification of ^13^C-SAM is given in section 2.2.3.1. Importantly, SAM has two enantiomers, but only (S,S) SAM is bioactive for the vast majority of enzymes. To ensure selective preparation of the (S,S) enantiomer, SAM is generated through enzymatic synthesis. Even so, SAM will readily racemize to the (R,S) form in aqueous solution unless precautions are taken. Here, we protect from racemization by performing the purification at 4 °C and storing the purified SAM at −80 °C in sulfuric acid. The sulfuric acid solutions typically favored in the literature and used here help to stabilize the SAM molecule against undesired decomposition, off-target methyl transfer, and (S,S) to (R,S) racemization. Due to the acidic nature of the final storage condition, which may cause downstream enzyme or substrate aggregation, add the cofactor to the buffering reagents and confirm the pH of the system immediately prior to the addition of protein substrate and enzyme.

**Fig. 4.**
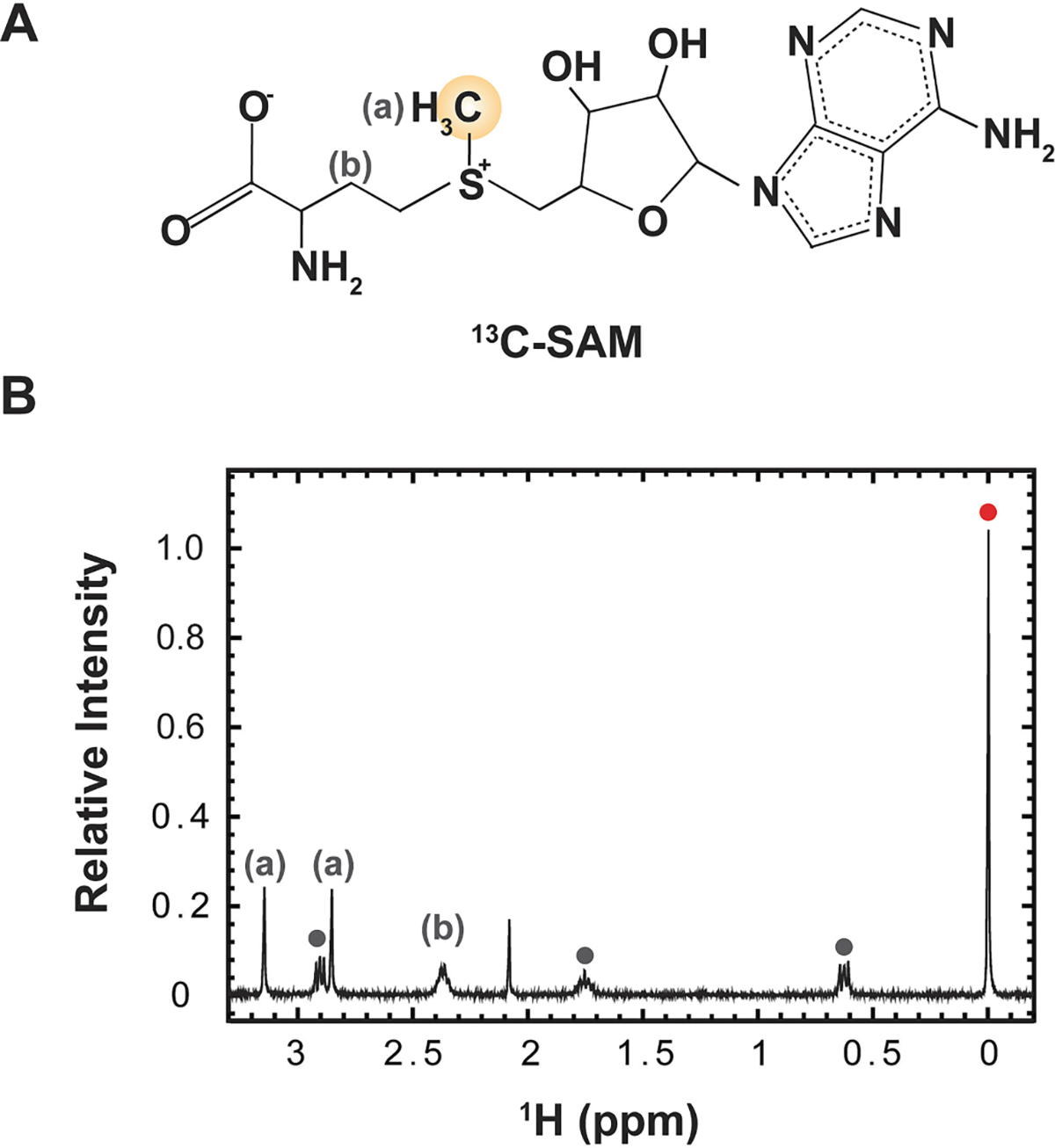
The isotopic enrichment scheme for ^13^C-methyl S-Adenosyl Methionine (SAM) and representative ^1^H-1D spectrum used to authenticate and quantify yield. (A) SAM is ^13^C enriched at the position encircled in yellow. (B) ^1^H-1D NMR spectrum of ^13^C-methyl SAM collected with Watergate water suppression and ^13^C-decoupling during acquisition. In acidic conditions used for storage, the methyl-attached protons appear at 3 ppm (labeled as ‘a’ in the spectrum and in panel A above; the CH_2_ resonance from position ‘b’ is also annotated). Peaks corresponding to DSS are notated with circles (BMRB entry bmse000795). The peak at 0 ppm (red circle) indicates the peak used for chemical shift referencing and quantitation. This ^1^H NMR spectrum is cropped for clarity; the full spectrum is displayed in Fig. S1.

^1^H NMR spectroscopy can be used to confirm that SAM is the dominant synthetic reaction product, assess SAM purity and racemization, and to quantify the SAM produced (Fig. 4, S1). SAM was identified by comparison to a ^12^C-SAM reference 1 H spectrum (HMDB0001185), noting that all SAM peaks shift downfield by ~ 0.1 ppm in the more acidic conditions used here to protect against racemization. Thus, the chemical shift observed after the final resuspension is 3.02 ppm. Note that in less acidic conditions ^1^H signals at 2.9 ppm arising from DSS will overlap with the ^13^C-SAM methyl proton singlet located at 2.93 ppm if ^13^C-decoupling is used, and peaks very near the water or acetate resonances (4.7 and 3.69 ppm, respectively) may be obscured due to intense solvent signals and the required use of solvent suppression techniques. Additionally, ^1^H NMR can be used to quantify ^13^C-SAM by relative integration of the SAM methyl protons to DSS methyl protons (0 ppm, BMRB entry bmse000795) as an internal standard. Similarly, ^1^H NMR reports on the quantity of (S,S) to (R,S) conversion through relative integration. During the purification process, we observe no detectable racemization (Fig. 4 and Fig. S1). We allowed racemization to occur at 4 °C over a period of days to provide a clear illustration of both resonance peaks, both for clarity and to evaluate the urgency of timely progression through the described protocol (Fig. S1). After an intentional delay of 12 days prior to completing the last purification step, the (R,S) SAM methyl proton peak at 2.99 ppm had 14% integration relative to the (S,S) SAM peak at 3.02 ppm. Additionally, the bioactive product is stable during long-term storage when kept at −80 °C as recommended. This provides a significant advantage in comparison with commercially available ^13^C-SAM, which in our experience tends to be received as a nearly 50/50 mixture of enantiomers. Strikingly, in the example provided, we observed only 40% of the ^13^C-SAM purchased was in the bioactive (S,S) form (Fig. S1). Thus, the time invested in performing the described ^13^C-SAM preparation method can yield pure, enantiospecific reagent, with excellent storage stability, in sufficient quantity for multiple downstream NMR experiments.

Both ^13^C_ali_, ^13^Cʹ-Acetyl CoA and ^12^C_ali_, ^13^Cʹ-Acetyl CoA are utilized as cofactors for enzymatic transfer of acetyl groups to H3 substrate shown here. The detailed protocol for synthesis is given in section 2.2.3.2. The synthetic protocols for generation of ^13^C_ali_, ^13^Cʹ-Acetyl CoA and ^12^C_ali_, ^13^Cʹ-Acetyl CoA are identical apart for the labeling scheme of the acetic anhydride starting material. Strategic enrichment of the starting cofactor provides versatility based on experimental priorities. Specifically, ^13^C_ali_, ^13^Cʹ-acetyl CoA cofactor permits ^13^C direct-detect without necessitating peptide substrate labeling, whereas ^12^C_ali_, ^13^Cʹ-Acetyl CoA permits access to experimental variants with shorter acquisition times.

**Fig. 5.**
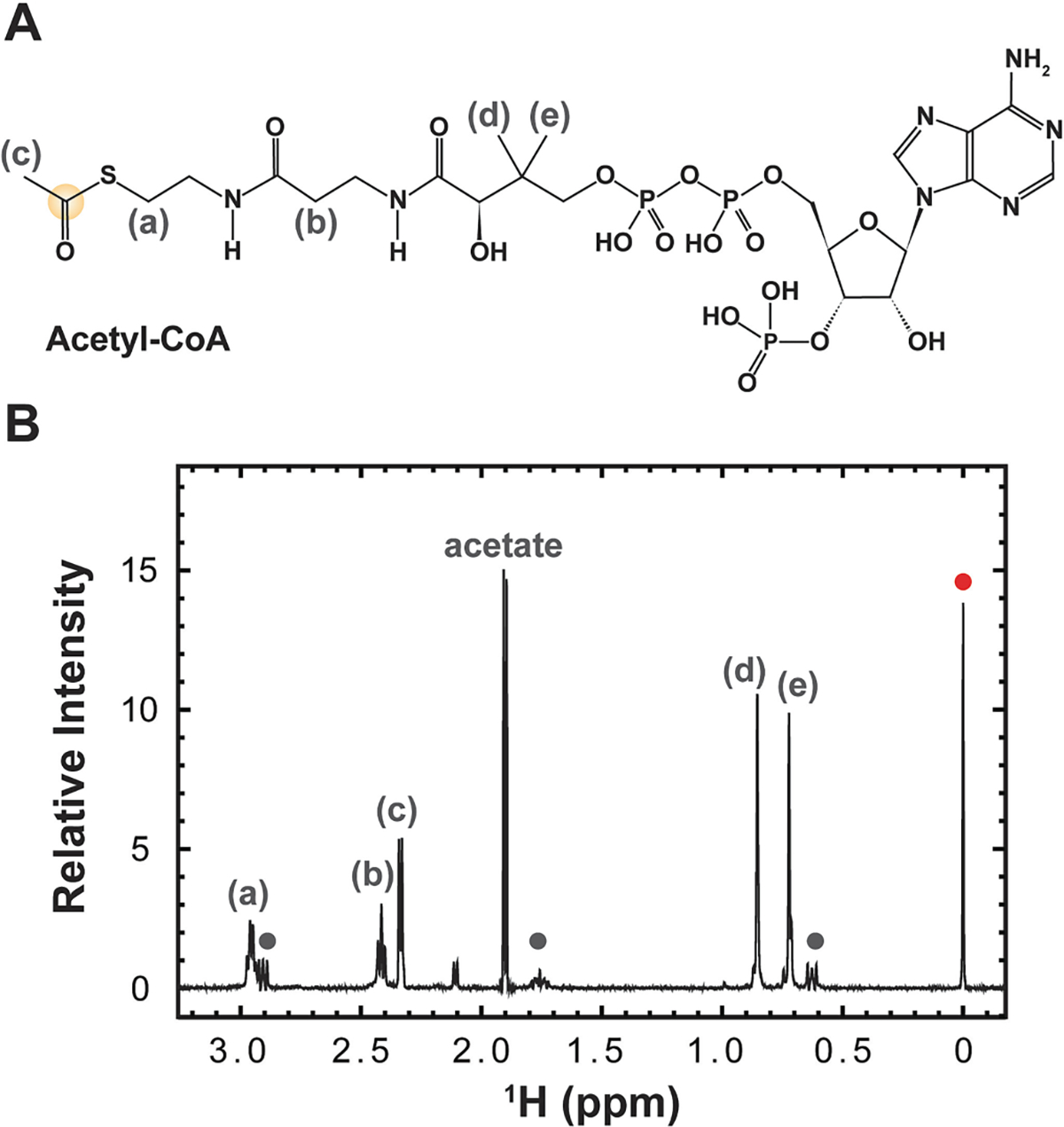
The isotopic enrichment scheme and representative ^1^H-1D spectrum used to authenticate and quantify ^12^C_ali_, ^13^Cʹ -acetyl CoA. (A) The chemical structure of acetyl CoA. The isotopically enriched carbon atom is encircled in yellow. (B)The product of ^12^C_ali_, ^13^Cʹ acetyl CoA synthesis was authenticated via 1D ^1^H NMR spectroscopy. The doublet centered at 2.3 ppm corresponds to the methyl group adjacent to the carbonyl group, labeled “c” (all acetyl CoA resonances included in this panel are labeled as a-e on the spectrum and in panel A). Peaks corresponding to DSS are notated with circles (BMRB entry bmse000795). The peak at 0 ppm (red circle) indicates the peak used for chemical shift referencing and quantitation. This ^1^H NMR spectrum is cropped for clarity; the full spectrum is displayed in Fig. S2.

The identity of acetyl CoA was confirmed by ^1^H NMR spectroscopy, using chemical shifts supplied by the CoA manufacturer (https://coalabio.com/acetyl-coa-nmr-spectrum.html) (Fig 5, S2). Note that in the case of both isotopic enrichment schemes, acetate, a side product of the synthetic reaction and degradation product of acetic anhydride, may be visible if not buffer exchanged from solution. Unremoved acetate can be visible in the [^1^H, ^13^C]-HSQC and [^13^C, ^13^C]-CaliCO-K_ac_ for ^13^C_ali_, ^13^Cʹ-Acetyl CoA.

### 3.2. [^1^H, ^13^C]-HSQC detection of ^13^C-methyl in modified lysine

Lysine mono-, di-, and tri-methylation using ^13^C-SAM as a cofactor and lysine acetylation using ^13^C_ali_, ^13^Cʹ-Acetyl CoA were previously observed by [^1^H, ^13^C]-HSQC NMR [23,24]. In the case of lysine methylation, the unique chemical shifts associated with mono-, di-, and tri-methylation are especially empowering because they permit species fractions of all three methylation levels to be simultaneously tracked. However, we do note that the representative data for methylation of H3K4 cannot address whether chemical shift degeneracy would impede analysis of systems in which multiple lysine residues are simultaneously modified. Further, [^1^H, ^13^C]-HSQC NMR was demonstrated as a viable strategy to monitor methylation kinetics in real time [23]. This strategy was made possible by short experimental times (<5.5 min) stemming from the sensitivity of the traditional ^1^H-detect spectroscopy. In general, [^1^H, ^13^C]-HSQC permits quantitative detection of PTM at concentrations as low as 1–50 μM [23]. [^1^H, ^13^C]-HSQC NMR was demonstrated as a viable technique to track a simultaneous enzymatic synthesis of acetyl CoA and acetyl transfer by ADA2/GCN5 to histone H3 tail [24]. In this case, H3K14 and H3K18 are simultaneously acetylated, but the resulting NMR resonances are degenerate, precluding individual analysis. It remains to be seen whether this is a general limitation or specific to the relatively homogeneous environment created by the sequence and structure of the H3 peptide.

**Fig. 6.**
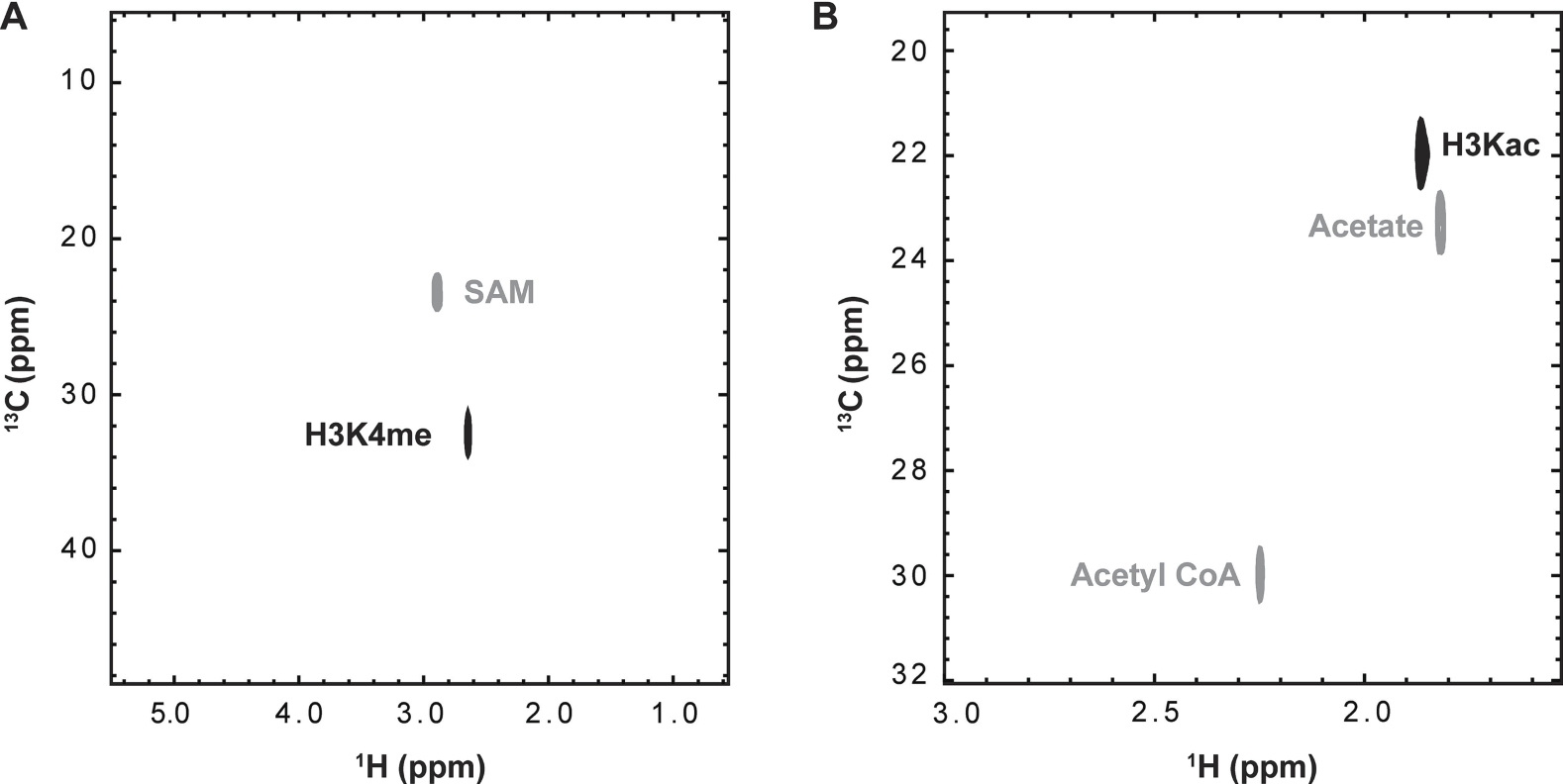
Traditional [^1^H, ^13^C]-HSQC detection of ^13^C-SAM, ^13^C_ali_, ^13^Cʹ -acetyl co-enzyme A, and the products of enzymatic lysine modification on H3 peptide facilitated by these cofactors. (A) A single peak arises from the methyl group of monomethyllysine (black) and is well resolved from the ^13^C-SAM resonance (grey). (B) A single peak arises from the methyl group on acetyllysine (black) and is well resolved from the starting material, ^13^C-Acetyl CoA, or the reaction byproduct, acetate (grey).

Representative [^1^H, ^13^C]-HSQC for both methylation and acetylation are shown here (Fig. 6). However, conventional ^1^H-detected experiments do face limitations. For example, analysis of these spectra can be impaired by spectral crowding arising from additives necessary to stabilize biological samples, such as organic co-solutes. In some cases, NMR spectral quality will be reduced by the influence of high salt concentrations on probe performance. Detection in more complex biologically derived matrix systems, such as blood plasma or cell lysate, or for *in vitro* studies of macromolecular crowding will face similar challenges from spectral complexity and potential influences on NMR probe performance. All of these difficulties can be abrogated through ^13^C direct-detect strategies [33], which motivates development of the experimental approaches central to the current protocols.

### 3.3. ^13^C direct-detect spectra of methyllysine

**Fig. 7.**
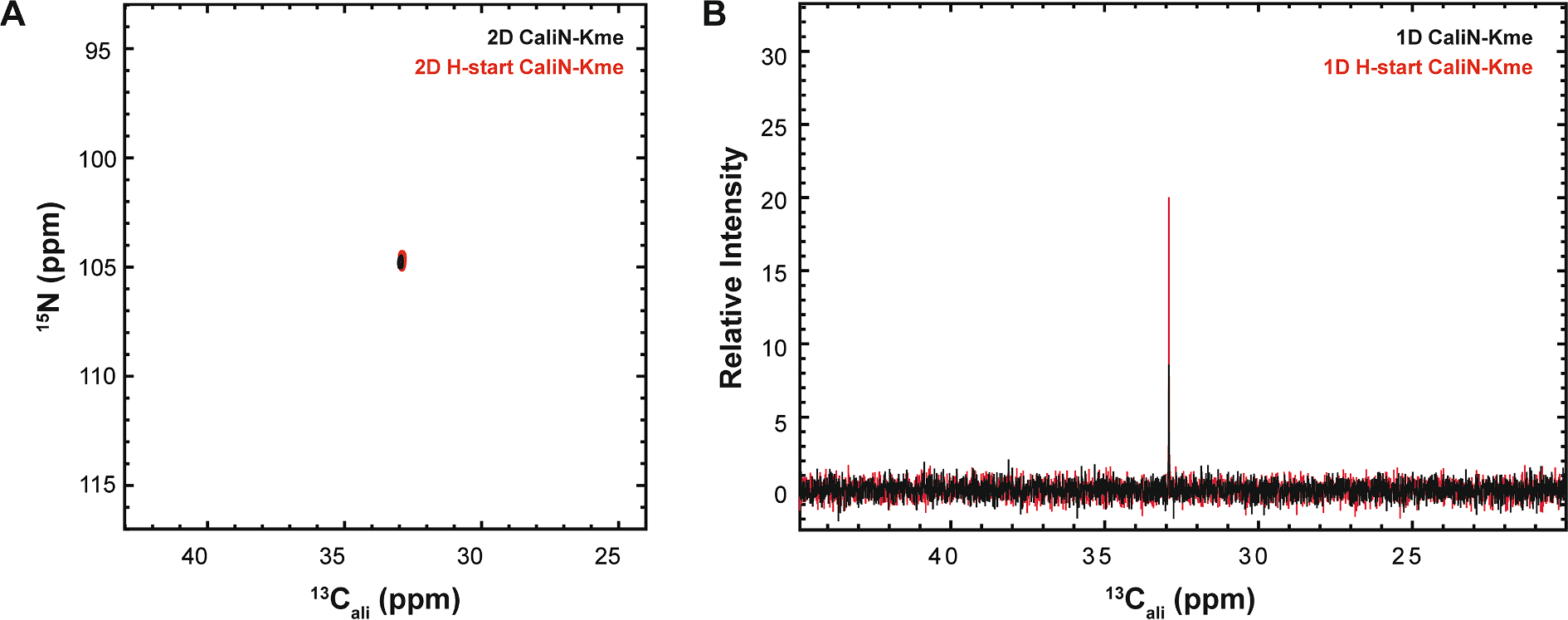
Optimization of methyl proton start step in the magnetization transfer pathway for the reported [^15^N, ^13^C]-CaliN-Kme. (A) A comparison of the carbon-start [^15^N, ^13^C]-CaliN-Kme (black) and the methyl proton-start [^15^N, ^13^C]-CaliN-Kme (red) shows preservation of high resonance quality in the 2D experiment. (B) In the 1D variant, the sensitivity enhancement (~2.7X) generated by adding the initial methyl proton magnetization transfer step is seen through the additional relative intensity of the red resonance.

Prior publications of methyllysine detection have all used ^1^H-detected HSQC techniques, motivating the demonstration here of a novel [^15^N,^13^C]-CaliN-K_me_ (Fig. 7), which monitors the correlation formed between the methyl carbon and lysine N_ε_ [23,34]. This experiment requires both ^13^C-enriched SAM and ^15^N-enriched peptide substrate. One obstacle that often limits applications of ^13^C direct-detect spectroscopy is the necessity for longer experimental time for signal averaging and/or greater substrate concentration, in comparison to comparable ^1^H-detected experiments. This arises from the inherently lower sensitivity of detection on the ^13^C nucleus due to its less-favorable gyromagnetic ratio. This known sensitivity problem can be partially alleviated by acquiring spectra on specialized ^13^C inner-coil probes, such as the Bruker TXO Cryoprobe, but as we demonstrate here taking this step is not strictly necessary. As an alternative to enhance the per-scan sensitivity of our ^13^C direct-detect spectra, we added a methyl ^1^H start element to the magnetization transfer pathways, resulting in a sensitivity enhancement of ~2.7X in comparison with the protonless experiment. In summary, current barriers for ^13^C direct-detect spectroscopy of methyllysine-containing proteins are surmountable and will diminish as this technology continues to mature, both on the hardware side and through evolution of protocols such as those described here.

### 3.4. ^13^C direct-detect spectra of acetyllysine

**Fig. 8.**
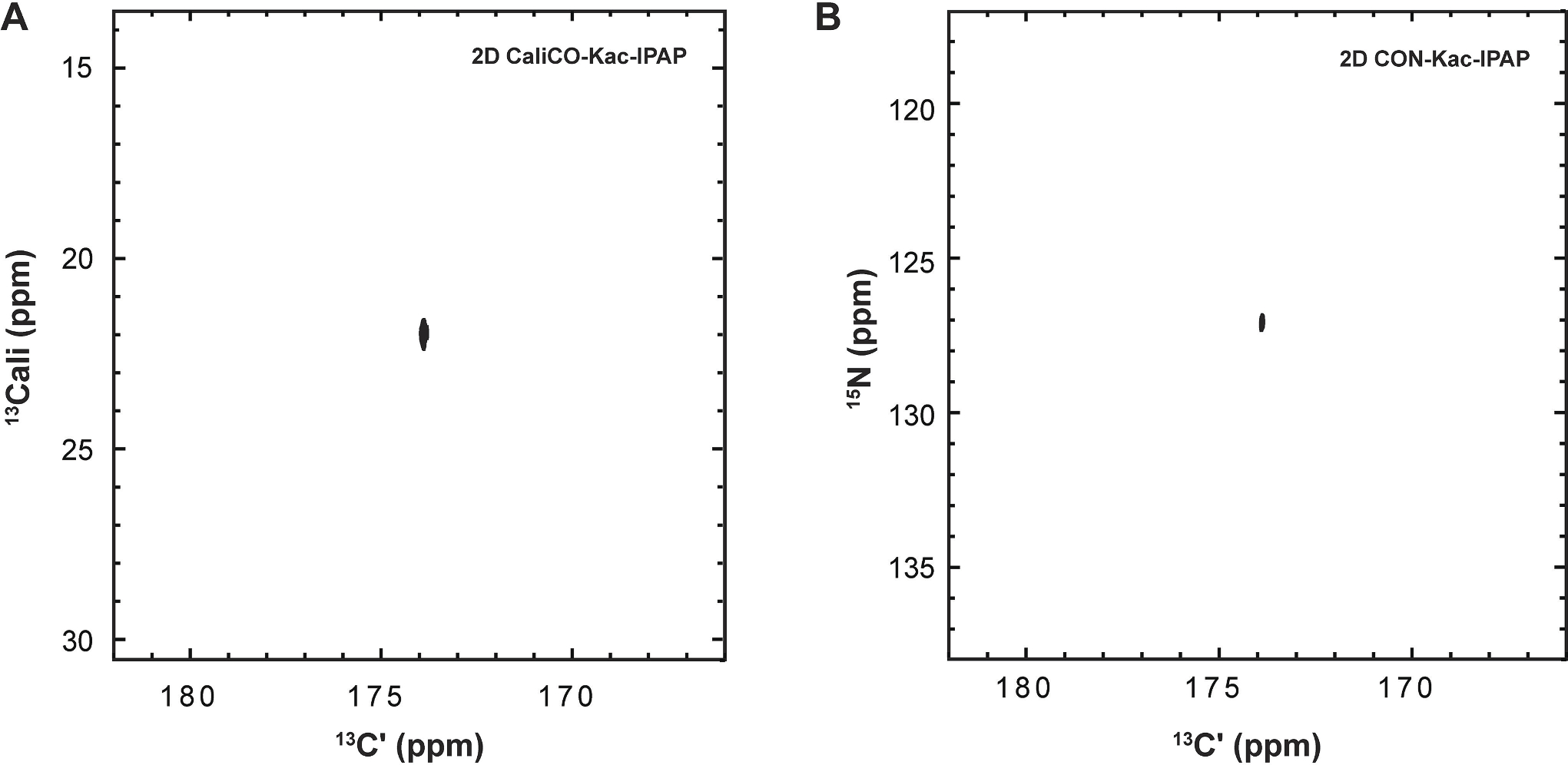
^13^C direct-detect NMR spectra of enzymatically generated ^13^C_ali_, ^13^Cʹ - acetyllysine. (A) The [^13^C,^13^C] CaliCO-Kac yields a correlation between the carbonyl carbon and the methyl carbon of the acetyl group without the need to isotopically label the peptide substrate. (B)The [^13^C,^15^N] CON-Kac-IPAP produces a correlation between the carbonyl carbon of the acetyl group and the lysine sidechain nitrogen therefore requiring ^15^N labeled protein substrate.

This work provides detailed protocols and expanded techniques for NMR detection of lysine acetylation. As previously reported, this technology leveraged ^13^C_ali_, ^13^Cʹ-Acetyl CoA as a cofactor, enabling the visualization of two correlations: between the aliphatic carbon and carbonyl carbon of the acetyl moiety or the carbonyl carbon and lysine-N_ε_ in the [^13^C, ^13^C]-CaliCO-K_ac_-IPAP and [^15^N, ^13^C]–CON-K_ac_-IPAP, respectively [24]. For completeness, representative spectra using these techniques are shown in Fig. 8. Carbon-start, methyl proton-start, and amide proton-start excitation schemes are available for both the [^13^C, ^13^C]-CaliCO-K_ac_-IPAP and [^15^N, ^13^C]–CON-Kac-IPAP (Table 2). Notably, the [^13^C, ^13^C]-CaliCO-K_ac_-IPAP does not require isotopic enrichment of the peptide substrate and benefits from 4-fold sensitivity enhancement with the addition of the methyl proton start excitation pathway. While the [^15^N, ^13^C]–CON-K_ac_-IPAP necessitates use of ^15^N-enriched peptide, this approach is of special interest because it records direct evidence that the novel carbon–nitrogen covalent bond has been formed, as the observed spectral correlation is brought into being upon PTM formation. The additional level of isotope filtering also offers advantages for background signal suppression, which can be leveraged to filter out co-solute signals and to suppress resonances from the backbone in uniformly enriched proteins.

**Fig. 9.**
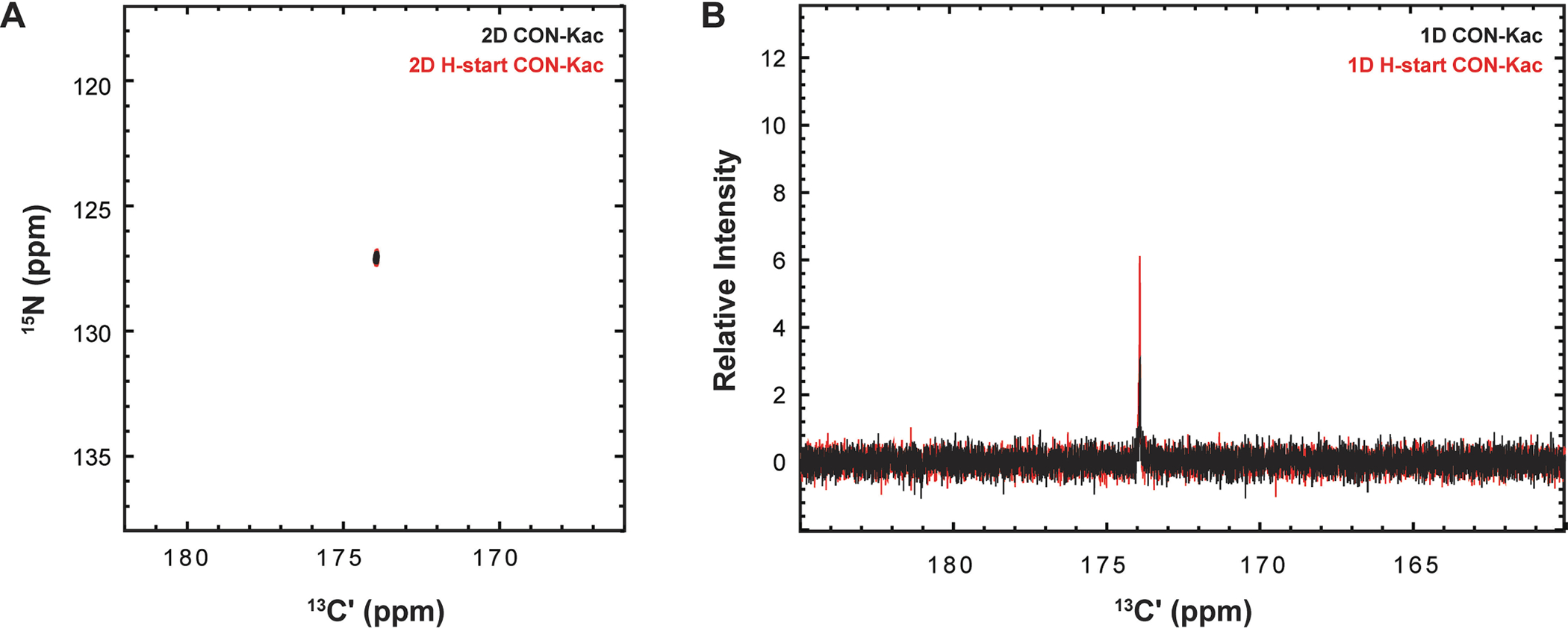
Optimization of amide proton start step in the magnetization transfer pathway for the reported [^15^N, ^13^C]–CON-Kac. (A) A comparison of the carbon-start [^13^C,^15^N]–CON-K_ac_ (black) and the amide proton-start [^13^C,^15^N]–CON-K_ac_ (red) shows preservation of high resonance quality in the 2D experiment. (B) In the 1D variant, the sensitivity enhancement (~2X) of the initial amide proton magnetization transfer step is seen through the additional relative intensity of the red resonance.

A known complication of ^13^C direct-detect NMR spectroscopy with uniform isotopic enrichment is the need to achieve homonuclear decoupling in the direct-detect dimension. In our prior work on acetyllysine NMR methods [24], we have chosen to solve this problem using in-phase anti-phase (IPAP) acquisition with virtual decoupling [31]. The IPAP virtual decoupling scheme is executed by collecting two interleaved experiments; as such, if the need for ^13^C-decoupling can be avoided, experimental acquisition time can be shortened by 50% before any other modifications are considered. Thus, to minimize time of acquisition, we strategically generated ^12^C_ali_, ^13^Cʹ-Acetyl CoA, eliminating the need for virtual decoupling. We observed acetylation of H3 tail using ^12^C_ali_, 13Cʹ -Acetyl CoA as a cofactor with the IPAPless [^15^N, ^13^C]–CON-K_ac_ (Fig. 9). To minimize sensitivity loss from detection on ^13^C, we added a ^1^H-start element to the magnetization transfer pathway, originating from the amide proton, resulting in an observed sensitivity enhancement of ~2X in comparison with the protonless experiment. In summary, users of these protocols now have access to a suite of acetyllysine-selective pulse programs, categorized in Table 2, and optimized for three distinct isotope labeling schemes that will support a range of biologically motivated applications.

### 3.5. 1D ^13^C direct-detect variants

A major contribution to experimental time in 2D NMR spectroscopy is the incremented delay necessary to collect chemical shift information in the indirect dimension. As the simplicity of the spectra reported here shows, the enhanced resolution of 2D spectroscopy is generally not needed for analysis of lysine modification, but rather it is the suppression of co-solvent signals from the requisite isotope filter in the experiments described here that provides the needed advantages. For example, traditional 1D proton and carbon spectra of methyllysine-containing H3 samples yielded spectra crowded with strong buffer and internal standard peaks (Fig. S3). Thus, for many applications it will be advantageous to leverage the isotope filters provided by the ^13^C direct-detect experiments in Table 1 and 2, but in a 1D format to further shorten acquisition time without loss of spectral resolution. Motivated by this logic, 1D variants of ^13^C direct-detect experiments for methyllysine and acetyllysine, based on the ^1^H-start [^15^N, ^13^C]-CaliN-K_me_ and amide ^1^H-start [^15^N, ^13^C]–CON-K_ac_ are demonstrated in Fig. 7 and Fig. 9, respectively. In addition to the sensitivity enhancements routinely seen in the ^1^H-start format, the shorter acquisition time required to gain full resolution that is inherent to 1D spectroscopy allows for detection of low concentration substrates (200 μM for methyllysine, 300 μM for acetyllysine at the time of writing (Fig. S4), with favorable collection times. While the lower limit of detection may still be too high to allow for robust kinetic applications, equivalent to those previously developed for ^1^H-detected observation of lysine methylation [23], this concentration regime is already low enough to support work with low solubility or low abundance peptide analytes.

## 4. Conclusion

The utilization of ^13^C-enriched cofactors to yield isotopically enriched methyllysine and acetyllysine for NMR analysis represents a significant advancement in biophysical research toward mechanistic understanding of these post-translational modifications (PTMs). This approach provides an opportunity to investigate methylation and acetylation without perturbation to native chemistry. Compared to existing NMR technologies, the use of ^13^C-enriched cofactors offers a simplified approach that is adaptable to a wide range of systems, while also providing an opportunity to conduct these studies without modifying the native chemistry of the modified proteins. Further enhancing the generality of this approach, the suite of NMR experiments described herein benefits from isotopic enrichment of substrate peptides, but does not require that this step be taken, thereby extending the applicability of this approach to less expensive synthesized peptides and proteins purified from mammalian cell extracts. Moreover, the implementation of ^13^C direct-detect strategies enables the detection of PTMs in complex biological matrices, as well as under harsh conditions such as high salt concentrations and the presence of organic co-solutes that otherwise complicate spectroscopy. Finally, the strategies presented here are readily adapted to 1D ^13^C direct-detect approaches (supported by strategic choices for isotopic labeling of acetyl CoA), which yield fast acquisition times while still maintaining the manifold benefits of ^13^C direct-detect techniques. With these improvements and the protocols for implementation described herein, we anticipate that this emerging technology will support high impact research in the lysine post-translational modification field, not only for the model case of histone tail modification, but across the broad range of protein substrates for lysine methyltransferase and lysine acetyltransferase enzymes that have been identified from proteomics, cellular, and biochemical assays.

## Supplementary Material

MMC1

## Figures and Tables

**Table 1:** Base experiments available for the detection of PTM utilizing ^13^C-cofactor.

Detection Method	PTM	Dimension	Isotopic Enrichment of Peptide Required	Isotopic Enrichment of Cofactor Required

[^1^H, ^13^C]-HSQC	Methylation [23] Acetylation [24]	2D	None	^13^C-SAM, ^13^Cali, ^13^C′ – Acetyl CoA, ^13^Cali, ^12^C′ – Acetyl CoA*
[^13^Cali, ^13^CO] – CAliCO-K_ac_	Acetylation [24]	2D	None (Note that ^15^N peptide is permitted)	^13^Cali, ^13^C′ – Acetyl CoA
[^15^N, ^13^C]-CaliN-K_me_	Methylation	2D, 1D	^15^N	^13^C-SAM
[^15^N, ^13^C]–CON-K_ac_, IPAP	Acetylation [24]	2D	^15^N (Note that ^13^C, ^15^N peptide is permitted)	^13^Cali, ^13^C′ – Acetyl CoA
[^15^N, ^13^C]–CON-K_ac_	Acetylation	2D, 1D	^15^N	^12^Cali, ^13^C′ – Acetyl CoA

* Although not demonstrated here, ^13^Cali, ^12^C′ - Acetyl CoA generated from 2,2′-^13^C_2_ acetic anhydride, (Cambridge Isotope Laboratories, CLM1160) will permit [^1^H, ^13^C]-HSQC but does not enable the ^13^C direct-detect methods described.

**Table 2:** 2D ^13^C direct-detect NMR experiments to monitor acetyllysine including variants. Bold font is used to designate carbon-start variants and sensitivity enhancement when adding magnetization transfer from either methyl or amide protons to the carbon-start variant are relative to the carbon-start experiment preceding it.

Experiment	Isotopic Enrichment Peptide	Isotopic Enrichment Cofactor	Sensitivity Enhancement

[^**13**^**C,** ^**13**^**C]-CAliCO-K**_**ac**_**-IPAP**	**Natural abundance (tolerates** ^**15**^**N)**	^**13**^**Cali**, ^**13**^**C′-Acetyl CoA**	**NA**
Amide start - CAliCO -K_ac_-IPAP	^15^N	^**13**^Cali, ^**13**^C′-Acetyl CoA	2.5
Methyl start - CAliCO-K_ac_-IPAP	Natural abundance (tolerates ^15^N)	^**13**^Cali, ^**13**^C′-Acetyl CoA	4
**[**^**13**^**C**, ^**15**^**N] CON-K**_**ac**_**-IPAP**	^**15**^ **N**	^**13**^**Cali**, ^**13**^**C′ - Acetyl CoA**	**NA**
Amide start-CON-K_ac_-IPAP	^15^N	^**13**^Cali, ^**13**^C′-Acetyl CoA	No enhancement
Methyl start- CON-K_ac_-IPAP	^15^N	^**13**^Cali, ^**13**^C′-Acetyl CoA	1/2
Methyl selective-CON-K_ac_-IPAP	^**13**^C/^**15**^N tolerated	^**13**^Cali, ^**13**^C′-Acetyl CoA	NA
**[**^**13**^**C**, ^**15**^**N] CON-K**_**ac**_	^**15**^ **N**	^**12**^**Cali,** ^**13**^**C′ - Acetyl CoA**	**NA**
Amide start- CON-K_ac_	^15^N	^**12**^Cali, ^**13**^C′-Acetyl CoA	2

## Data Availability

Data will be made available on request.
