# Supplementary material for: Advances in direct detection of lysine methylation and acetylation by nuclear magnetic resonance using 13C-enriched cofactors
Source: Methods. Author manuscript; Available in PMC 2023 Oct 1. (PMC10528339; doi:10.1016/j.ymeth.2023.07.010)
Supplement: MMC1 [file NIHMS1923925-supplement-MMC1.pdf]

Supplementary Information

Advances in Direct Detection of Lysine Methylation and Acetylation by Nuclear Magnetic Resonance using ^{13}C-Enriched Cofactors

Olivia A. Fraser[1], Kevin E. W. Namitz[2], Scott A. Showalter[1,2*]

[1]Center for Eukaryotic Gene Regulation, Department of Biochemistry and Molecular Biology, The Pennsylvania State University, University Park, PA 16802
[2]Department of Chemistry, The Pennsylvania State University, University Park, PA 16802

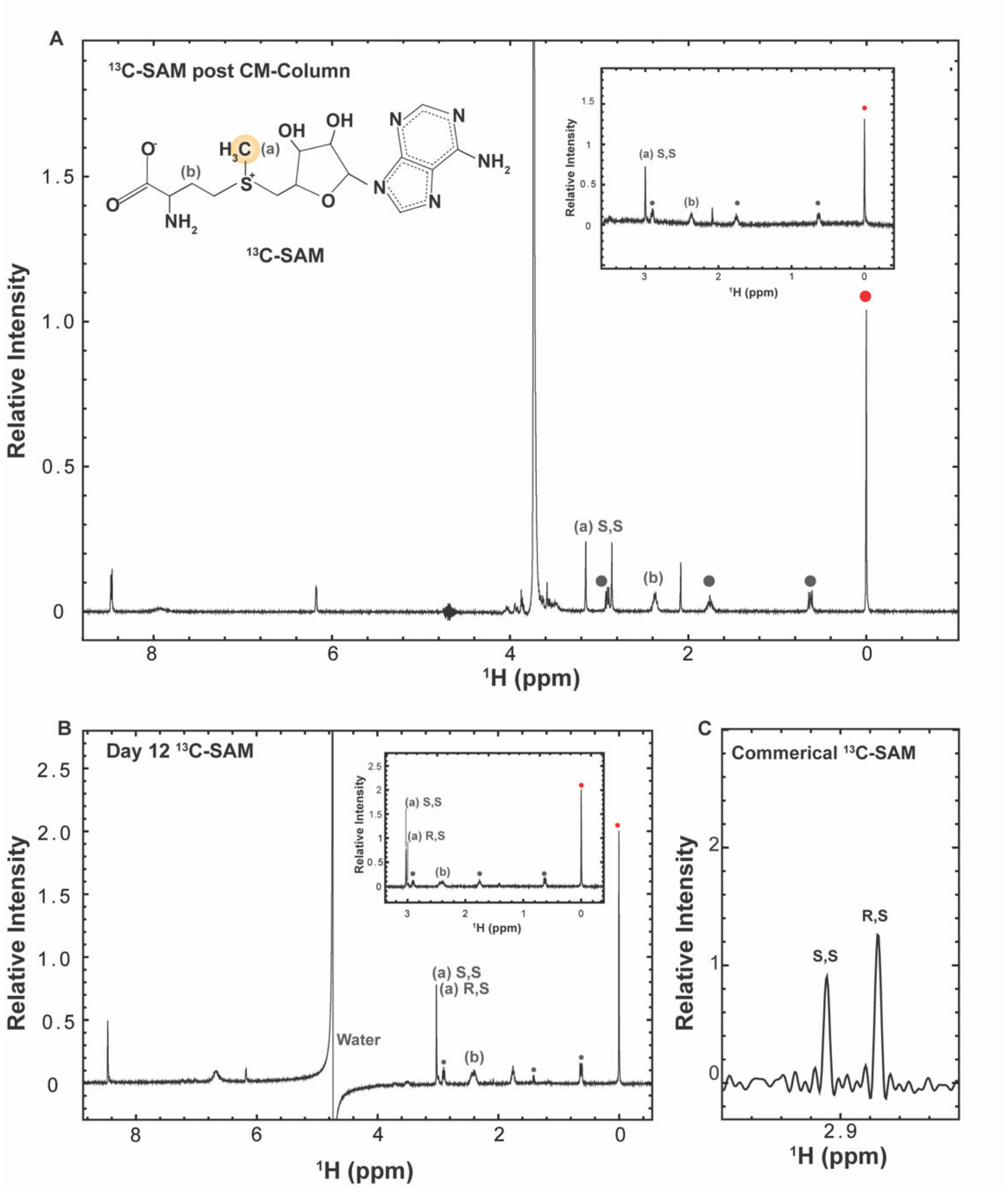

Figure S1. ^{1}H 1D NMR characterization of ^{13}C SAM: immediately after the first step of purification (A), after 12 days of allowing racemization to proceed (B), and commercially purchased (C). In panel (A), the spectrum corresponding to Fig. 4 without zoom is displayed. This spectrum was acquired immediately after collection from the CM column described in section 2.2.3.1. As the spectrum here was acquired with no ^{13}C decoupling, the (S,S) SAM peak presents as a doublet, labeled (a). To aid in comparison with panel (B), a zoom of a spectrum collected on the identical sample using ^{13}C-decoupling is displayed in the inset. Note that a peak corresponding to (R,S)-SAM is not visible in either spectrum. (B) A ^{1}H 1D spectrum with ^{13}C decoupling and Watergate water suppression, acquired 12 days after completing purification with storage at 4 °C. (C) A 1D projection from a [^{1}H, ^{13}C]-HSQC acquired from commercially purchased ^{13}C-SAM.

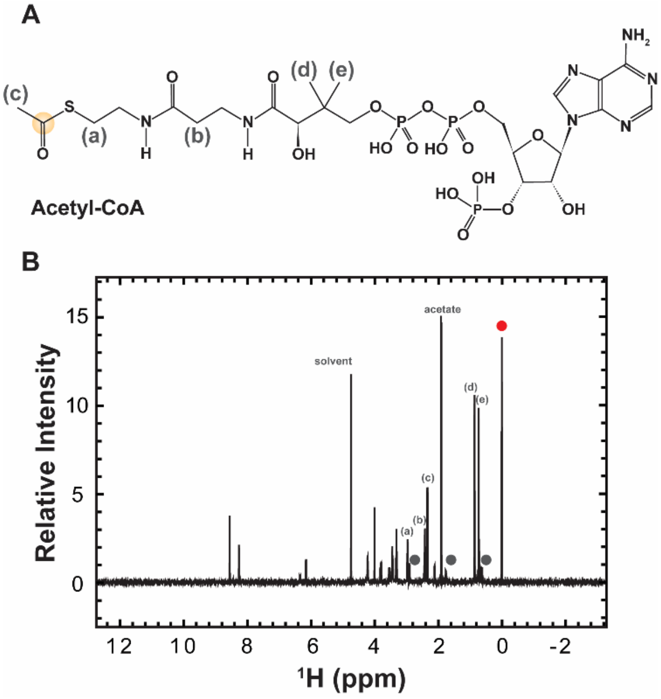

Figure S2. The isotopic enrichment scheme and representative ^{1}H-1D spectrum used to authenticate and quantify $^{12}C_{ali}$, $^{13}C'$ -acetyl CoA, corresponding to Fig. 5 in the main text. (A) The chemical structure of acetyl CoA. The isotopically enriched carbon atom is encircled in yellow. (B) The spectrum used for authentication of $^{12}C_{ali}$, $^{13}C'$ -acetyl CoA. DSS peaks are annotated by circles, and those peaks corresponding to acetyl CoA protons shown in Fig. 5 are likewise annotated here by letters (a) – (e).

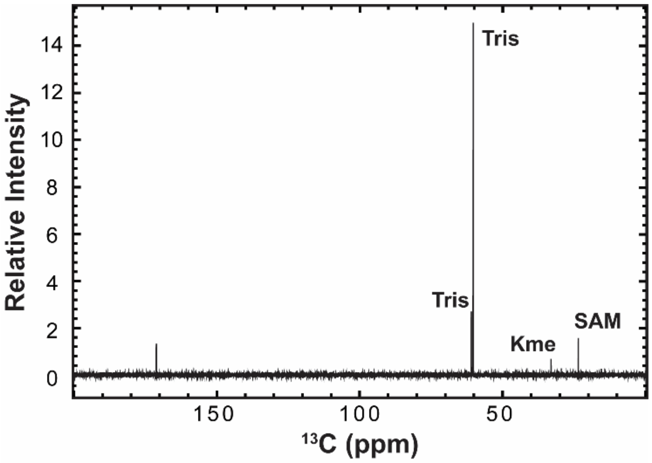

Figure S3. ^{13}C 1D experiment on monomethylated H3 (100 μM) where the peak arising from methyllysine is seen at 33 ppm (labeled Kme). However, the monomethyllysine peak has relatively low intensity in comparison to peaks arising from the matrix (Tris buffer) or reaction precursor, ^{13}C-SAM, as it lacks the selectivity of the [^{15}N, ^{13}C]-CaliN-Kme variants.

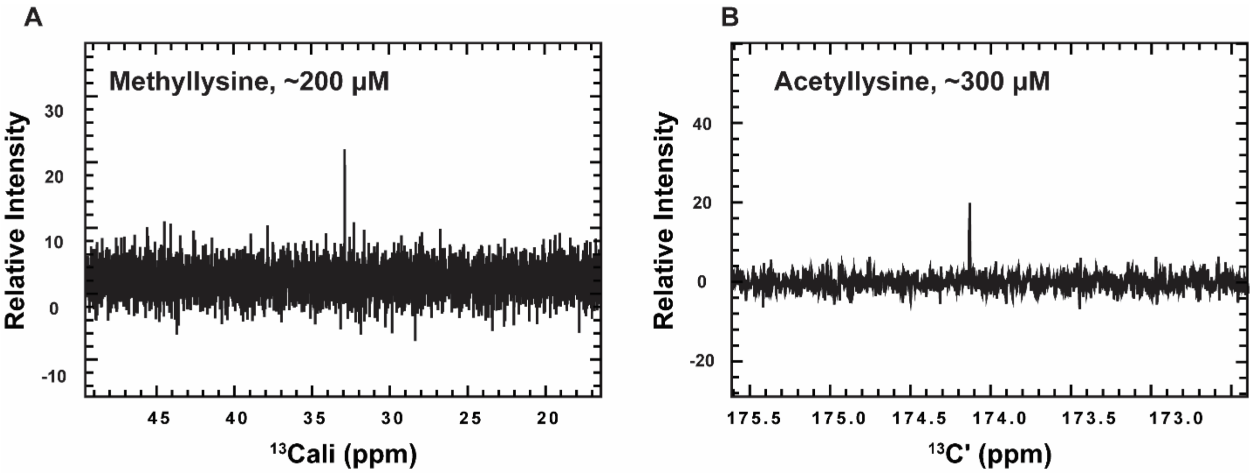

Figure S4. A presentation of the 1D proton-start ^{13}C direct-detect experiments, the [^{15}N, ^{13}C]-CaliN-K_{me} and [^{15}N, ^{13}C]-CON-K_{ac}, at the lowest concentrations we observed signal.
